# Critical Evaluation of “GLP‐1 and Dual GLP‐1/GIP Receptor Agonists in Heart Failure With Mildly Reduced or Preserved Ejection Fraction: A Systematic Review and Meta‐Analysis”

**DOI:** 10.1002/clc.70249

**Published:** 2025-12-26

**Authors:** Zubaida Bibi

**Affiliations:** ^1^ Ayub Medical College Abbotabad Abbotabad Pakistan

Dear Editor,

1

I read with great interest the correspondence “GLP‐1 and Dual GLP‐1/GIP Receptor Agonists in Heart Failure With Mildly Reduced or Preserved Ejection Fraction: A Systematic Review and Meta‐Analysis” by Ahmed et al. examining the role of GLP‐1 receptor agonists (GLP‐1 RAs) and dual GLP‐1/GIP receptor agonists in patients with heart failure with mildly reduced or preserved ejection fraction (HFmrEF/HFpEF [1]). The authors should be acknowledged for their timely and rigorous synthesis of contemporary randomized data, contributing meaningfully to an important therapeutic gap in HFpEF. Despite these strengths, several limitations inherent to the available evidence deserve emphasis to appropriately contextualize the findings.

First, although all included studies were randomized, many HFpEF outcomes were obtained from post‐hoc or secondary analyses of trials mainly designed for obesity, diabetes, or broad cardiovascular risk reduction (e.g., SELECT, FLOW, and EXSCEL). Such analyses are informative but are not well powered for HFpEF‐specific endpoints and may be impacted by variations in heart‐failure diagnosis and adjudication, thereby limiting causal inference despite randomization [2].

Second, care should be taken when interpreting the lack of a statistically significant effect on cardiovascular or all‐cause mortality. The pooled analysis appears underpowered to identify mortality differences, given relatively short follow‐up periods and low absolute numbers of deaths in several contributing trials, including SUMMIT. In HFpEF, mortality benefits often require longer follow‐up and larger event numbers to become apparent [3, 4].

Third, the generalizability of these findings is limited by the obesity dominant nature of the study populations, with mean body‐mass indices consistently above 34 kg/m². Since HFpEF is a heterogeneous syndrome, the observed benefits likely apply mainly to obesity‐related or metabolically driven HFpEF. However, extrapolation to lean or non‐metabolic HFpEF phenotypes should be undertaken cautiously [5, 6].

In summary, this meta‐analysis provides valuable evidence that incretin‐based therapies may be a promising adjunctive strategy for selected patients with HFpEF, especially those with obesity and metabolic dysfunction. However, the above limitations highlight the need for prospective, HFpEF‐specific randomized trials with broader follow‐up and diverse phenotypic representation to clarify the role of these agents across the HFpEF spectrum.

## Funding

The author received no specific funding for this work.

## Consent

The author has nothing to report.

## Conflicts of Interest

The author declares no conflicts of interest.

## Data Availability

No new data was created or generated in this work.
